# Investigating cardiac genetic background in sudden infant death syndrome (SIDS)

**DOI:** 10.1007/s00414-024-03264-6

**Published:** 2024-06-07

**Authors:** Francesca Cazzato, Mònica Coll, Simone Grassi, Anna Fernàndez-Falgueras, Laia Nogué-Navarro, Anna Iglesias, Josep Castellà, Antonio Oliva, Ramon Brugada

**Affiliations:** 1 Department of Health Surveillance and Bioethics, Section of Legal Medicine, Fondazione Policlinico A. Gemelli IRCCS, Università Cattolica del Sacro Cuore, 00168 Rome, Italy; 2 Cardiovascular Genetics Centre, University of Girona-IDIBGI, 17190 Salt, Spain; 3 Department of Health Sciences, Section of Forensic Medical Sciences, University of Florence, Largo Brambilla 3, 50134 Florence, Italy; 4 Cardiology Department, Hospital Universitari Doctor Josep Trueta, 17003 Girona, Spain; 5 Faculty of Medicine, University of Vic—Central University of Catalonia (UVic-UCC), Vic 08500 Can Baumann, Spain; 6 Forensic Pathology Service, Institut Medicina Legal Ciències Mèdiques Catalunya, Barcelona, Spain; 7 Centro de Investigación Biomédica en Red de Enfermedades Cardiovasculares (CIBERCV), 28029 Madrid, Spain; 8 Medical Science Department, School of Medicine, University of Girona, 17003 Girona, Spain

**Keywords:** Sudden Infant Death Syndrome, Sudden Cardiac Death, Post-mortem genetic testing, Forensic autopsy

## Abstract

Sudden infant death syndrome (SIDS) is still the leading cause of death for newborns in developed countries. The pathophysiological mechanisms have not been fully clarified, but in some of SIDS cases variants of genes associated with inherited cardiac conditions are found. In this study, an analysis of SCD-related genes was performed to determine the prevalence of rare pathogenic (P) or likely pathogenic (LP) variants that could provide an unambiguous explanation for the fatal event. A cohort of 76 SIDS cases underwent Next-Generation Sequencing (NGS) analysis with a custom panel of SCD-related genes. Rare variants were classified according to the guidelines provided by the American College of Medical Genetics and Genomics (ACMG) and the specifications of the ClinGen association. Post-mortem genetic testing identified 50 (65.8%) carriers of at least one variant in SCD genes. 104 rare genetic variants were found, 65.4% in genes encoding structural proteins. Only 4 out of 76 cases (5.3%) hosted at least a P or LP variant found in genes with structural or structural/arrhythmogenic functions (*SLC22A5, SCN5A, MYL3*and *TTN*). 99 variants were classified as of uncertain significance (VUS). The difference in the distribution of variants between gene groups by function was not statistically significant (chi square, p = 0,219). Despite this, most of the variants concerned structural genes that were supposed to have a close interaction with ion channels, thus providing an explanation for the arrhythmic event. Segregation analysis, reclassification of VUS variants and identification of new associated genes could clarify the implications of the current findings.

**Supplementary Information:**

The online version contains supplementary material available at 10.1007/s00414-024-03264-6.

## Introduction

Sudden unexpected infant death (SUID) encompasses any unexpected fatal event occurred during infancy, thus covering both explained and unexplained deaths [1, 2]. When the full case investigation—including the examination of the death scene, complete autopsy, histopathological and toxicological analysis and review of the medical history- is inconclusive, it is referred to as “sudden infant death syndrome” (SIDS) [3, 4]. The term SIDS is used for unexpected and unexplained deaths of infants < 1 year old that usually occur during sleep [1, 5]. Although the incidence has been progressively reduced over the last 30 years due to public health prevention campaigns and improved standardized classification of pediatric deaths, SIDS still is the leading cause of death in this age group in developed countries [5].

Despite this, to date, the pathophysiological mechanisms underlying SIDS have not been fully clarified. Traditionally, the pathogenesis of SIDS has been explained by the multifactorial “triple risk model”, resulting from the overlapping of critical developmental period, an exogenous stressor and an underlying intrinsic vulnerability [6]. In 2022, Harrington et al. demonstrated an altered cholinergic homeostasis in SIDS cases compared with controls and proposed Butyrylcholinesterase specific activity (BChEsa) as a biochemical marker of increased measurable vulnerability in live infants [7].

It has been hypothesized that the genetic background, consisting of rare variants in genes associated with hereditary heart disease, inherited metabolic conditions, central nervous system regulation, immune dysfunction and nicotine response, plays a causal role in SIDS [5, 7, 8]. In the last few years, certain genetic variants in cardiac structural proteins and ion channels in the adults have been proposed as a monogenic cause of death in some SIDS cases [5, 6, 9–11]. Nowadays, post-mortem genetic testing is a widely accepted diagnostic tool during a comprehensive forensic investigation in sudden death (SD) cases without a conclusive cause of death [12, 13]. In SIDS cohorts it may provide information on the etiopathogenetic mechanisms underlying the syndrome, suggesting in some cases further analysis in infant’s first-degree relatives to predict and prevent the risk of sudden cardiac death (SCD) in the family. However, careful interpretation of the pathogenicity of genetic variants is required before translation into clinical practice [10]. A joint consensus of the American College of Medical Genetics and Genomics (ACMG) and the Association for Molecular Pathology (AMP) suggest a comprehensive evaluation including predictive functional testing (in vitro, in silico, in vivo), family segregation analysis and genotype–phenotype correlations by a multidisciplinary team of experts [12, 14].

In the current study, a comprehensive Next Generation Sequencing (NGS) analysis of SCD-related genes was performed in 76 SIDS cases. The aim of this investigation was to assess whether cardiac genetic predisposition can provide an unambiguous explanation for the fatal event. The purpose was also to identify the type of genes involved to elucidate the prevalent underlying mechanisms of sudden cardiac death (SCD) in SIDS according to the current classifications and scientific literature review.

## Materials and methods

### Forensic investigations

76 apparently healthy infants younger than 1-year-old who suddenly died between 2012 and 2021 without a clear cause of death underwent a full forensic examination performed at the Institute of Legal Medicine of Catalonia (IMLC).

Forensic analyses were performed according to the international recommendations, tailored with the specific recommendations issued by the Institute of Legal Medicine of Catalonia (IMLC) for investigation of sudden unexpected death in infants [15, 16]. Post-mortem examinations included a full autopsy with tissues and biological fluids collection for histopathological, toxicological, microbiological and genetic analyses.

The study was approved by the ethical committee of University Hospital of Girona “Doctor Josep Trueta” (Spain) (ethical approval reference number: 2011031) and complies with the ethical guidelines of the Declaration of Helsinki 2008.

### Genetic analysis

Post-mortem genetic testing was performed in all infants with an undetermined cause of death after inconclusive forensic investigations. The genetic analysis was carried out to assess the existence of genetic variants related to arrhythmic and/or structural heart diseases associated with SCD.

Genomic DNA from post-mortem whole blood was extracted with Chemagic MSM I (PerkinElmer, Waltham, MA, USA). DNA concentration was determined using the Qubit fluorometer (Thermo Fisher Scientific, Waltham, MA, USA), and 3 μg of DNA was used for library preparation. NGS analysis was performed using a custom resequencing panel of selected genes related with SCD, designed and optimized by our own group of bioinformatics and enriched using the SureSelect Custom Target Enrichment System Kit following the manufacturer’s instructions (Agilent Technologies, Santa Clara, CA, USA). The paired-end sequencing process was carried out on MiSeq System (Illumina, Inc., San Diego, CA, USA) using 2 × 76-basepair read length. DNA reads were mapped to an annotated reference sequence to then determine the extent of variation.

Population data were obtained from Genome Aggregation Database (gnomAD; http://gnomad.broadinstitute.org, accessed on 22 May 2022) and all variants with a variant allele frequency (VAF) < 0,1% in Popmax Filtering were considered rare. In *silico* prediction tools such as PolyPhen2 [17], Provean [18] and Mutation Taster [19] were consulted to predict pathogenicity of rare genetic variants. Finally, we also used the AutoPVS1 (http://autopvs1.genetics.bgi.com/) for interpretation of null variants.

Sanger sequencing was performed when the coverage was lower than 30X and to validate rare variants (VAF < 0,1%). Hence, polymerase chain reaction (PCR) was performed, and after a purification through ExoSAP-IT (USB Corporation, Cleveland, OH, USA) the product was directly sequenced using the dideoxy chain-termination method in an ABI Prism Big Dye® Terminator v3.1 Cycle Sequencing Kit (Applied Biosystems, Waltham, MA, USA). Sequencing was performed using a 3500 Genetic Analyzer (Applied Biosystems, Waltham, MA, USA) and analyzed by SeqScape Software v2.5 (Life Technologies, Waltham, MA, USA). Genetic variants were reported in compliance with the recommendations given by the Human Genome Variation Society (HGVS).

Moreover, Human Gene Mutation Database (HGMD) (http://www.hgmd.org) and ClinVar (https://www.ncbi.nlm.nih.gov/clinvar/intro/) were consulted to check for pathogenic variants previously reported in the scientific literature. All variants were classified as pathogenic (P), likely pathogenic (LP), or variants of unknown significance (VUS) according to the pathogenicity items provided by the guidelines for the interpretation of sequence variants of the American College of Medical Genetics and Genomics (ACMG) and the specifications of the ClinGen association [14].

PM2_Supporting item in the ACGM classification was considered then fulfilled with VAF < 0.004% for cardiomyopathies and < 0.001% for channelopathies.

Finally, all rare variants (VAF < 0,1%) were classified into three groups according to the function and related disease of the encoded protein: 1) Structural: variants in genes related to cardiomyopathies; 2) Arrhythmogenic: variants in genes related to channelopathies; 3) Both: variants in genes related both to cardiomyopathies and channelopathies.

### Data collection

Data were registered in the research electronic data capture (REDCap) tools hosted at Institut Català de la Salut (ICS). REDCap is a secure web application designed to support online surveys/databases for research studies providing an intuitive interface for data capture, multi-site access for data managing/tracing and automatized export procedures for data downloads to common statistical packages [20, 21].

### Statistical analysis

A descriptive analysis of the characteristics (both demographic and clinical) of the patients was performed. Continuous data were summarized as the mean (with related standard deviation [SD]) and counts and percentages were used to report categorical data. Probability values for categorical parameters were determined using Pearson chi-square test. The IBM SPSS Statistics package v. 26.0 was used for statistical analysis. A *p* value of less than or equal to 0.050 was set as the cutoff for statistical significance.

## Results

The cohort consisted of 76 apparently healthy infants < 1 year old (32 males, 44 females; average age 0.56 ± 0.42 years) who suddenly died between 2012 and 2021 without a clear cause of death after full forensic examination/complete case investigation and with a genetic test performed.

The average weight and height were respectively 5756 ± 2622 g (6552 ± 3031 g in males and 5183 ± 2156 g in females) and 60.7 ± 16.5 cm (66.5 ± 21.9 cm in males and 56.0 ± 8.2 cm in females).

The post-mortem genetic analysis and the interpretation of the results according to ACMG recommendations focused on variants with the highest degrees of pathogenicity at classification. Post-mortem genetic testing revealed 50 (65.8%) carriers of at least a variant in the selected genes related to SCD, while in 26 (34.2%) cases no variants were identified in the tested genes. Carriers of rare variants were equally distributed between males (n = 21; 42%) and females (n = 29; 58%). Therefore, in 50 genetic carriers, 104 rare genetic variants were identified and all of them were heterozygous. Hence, 21 samples had 1 variant (42%), 13 samples had 2 variants (26%), 10 samples had 3 variants (20%), 4 samples had 4 variants (8%), 1 sample had 5 variants (2%) and 1 sample had 6 variants (2%).

104 SCD-related variants were classified into three groups: pathogenic (P), likely pathogenic (LP) and variants of uncertain significance (VUS). Pathogenicity assessment is used to predict a possible correlation between genotype and phenotype (SIDS). Only 1 (1.0%) was classified as pathogenic (P), 4 (3.8%) as likely pathogenic (LP), and 99 (95.2%) as variants of uncertain significance (VUS). Finally, in 4 out of 76 cases (5.3%) at least one pathogenic (P) or likely pathogenic (LP) variant could be considered as a monogenic cardiac cause responsible for the sudden death of the infant.

### Pathogenic (P) and likely pathogenic (LP) variants

The only pathogenic variant was the *missense SLC22A5*_c.845G > A, causing a change of Arginine (Arg, R) -charged, basic to Glutamine (Gln, Q) -neutral, polar (Tab. 1). This variant has already been reported in the HGMD and ClinVar databases in patients affected by Renal carnitine transport defect disease with an autosomal recessive pattern of inheritance. The four likely pathogenic variants were the *missense* variant *SCN5A*_ c.5051_5052delinsTT inducing a change of Tryptophan (Trp, W) -charged, acidic to Phenylalanine (Phe, F) -neutral, non-polar; the *missense* variant *MYL3_*c.466G > T causing a change of Valine (Val, V) -neutral, non-polar to Leucine (Leu, L) -neutral, non-polar; the *nonsense* variant *TTN*_ c.67439 T > A causing a STOP codon in p.(Leu22480*); the single nucleotide *intronic* variant *TTN*_ c.32705-1G > A (Tab. 1). All these four LP variants were classified of uncertain/-likely pathogenic significance in ClinVar. Two of them have been previously reported in people diagnosed with Dilated cardiomyopathy (DCM) (*TTN*_ c.67439 T > A; *TTN*_ c.32705-1G > A), 1 in patients with Brugada syndrome (BrS) (*SCN5A*_ c.5051_5052delinsTT) and 1 in individuals affected by Hypertrophic cardiomyopathy (HCM) (*MYL3_*c.466G > T).

**Table 1:** Data of P and LP genetic variants identified. ACMG, American College of Medical Genetics and Genomics; AF, Allele Frequency; BrS, Brugada Syndrome; DCM, Dilated Cardiomiopathy; GnomAD, Genome Aggregation Database; HCM, Hypertrophic Cardiomyopathy; NA, Not Available. *The context referred to variant-associated diseases previously reported in the scientific literature via* the HGMD and ClinVar databases*

Variant Description		GnomAD Database	In silico prediction tools			Variant Classification	
Gene (isoform)	Variant (cDNA and Protein)	Popmax Filtering AF (%)	PolyPhen2	PROVEAN	Mutation Taster	ACMG score	Context*
*SCN5A* (NM_198056.2)	c.5051_5052delinsTT p.(Trp1684Phe)	NA	Possibly damaging	Not Available	Disease causing	LP	BrS
*SLC22A5* (NM_003060)	c.845G > A p.(Arg282Gln)	0.0003976	Probably damaging	Deleterious	Disease causing	P (autosomal recessive pattern of inheritance)	Renal carnitine transport defect
*MYL3* (NM_000258)	c.466G > T p.(Val156Leu)	0.000701	Possibly damaging	Deleterious	Disease causing	LP	HCM
*TTN* (NM_133378)	c.67439 T > A p.(Leu22480*)	NA	NA	NA	NA	LP	DCM
*TTN* (NM_133378)	c.32705-1G > A	0.0031924	No splicing effect	No splicing effect	No splicing effect	LP	DCM

### Variants of uncertain significance (VUS)

99 rare variants were classified as variants of uncertain significance (VUS). 91 (91.9%) were *missense* variants of *ACTN2, AKAP9, ANK2, BAG3, CACNA1C, CACNA1H, CASQ2, COL3A1, DSG2, DSP, EMD, FBN1, FBN2, FHL2, FLNA, FLNC, HCN4, JPH2, KCNE1, KCNH2, KCNQ1, LAMA4, MYH11, MYH6, MYH7, NUP155, PDLIM3, PRKAG2, RYR2, SCN10A, SCN5A, TGFBR3, TMPO, TRIM63, TRPM4* and *TTN* genes. 4 (4.1%) were single nucleotide variants in intronic regions of *PRKAG2, CACNB2, FBN1* and *TRDN* genes. Finally, 2 (2.0%) were *nonsense* variant in *SCN1B* and *LAMA4*, 1 (1.0%) an *in-frame* deletion of *NEXN*, and 1 (1.0%) a small deletion in the intronic region of *MYH6*.

### Variants distribution

**Fig. 1:**
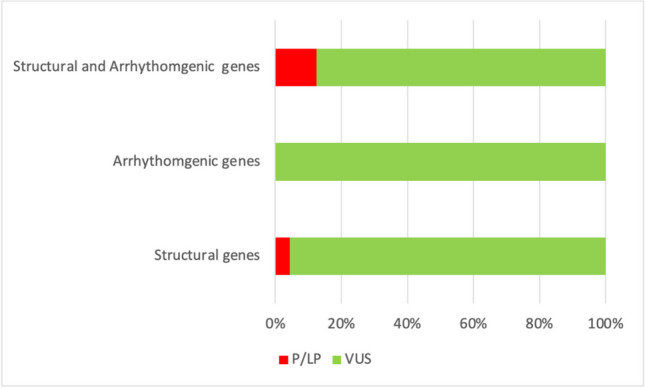
Percentage of P/LP variants (in *red*) and VUS (in green) sorted by type of gene affected (i.e., structural genes, arrhythmogenic genes and structural and arrhythmogenic genes)

In 50 genetic carriers, 104 rare genetic variants were identified: most of them concerned structural genes with 68 of 104 (65.4%) rare variants, then 20 (19.2%) variants in arrhythmogenic genes and 16 (15.4%) in genes involved in both structural and arrhythmogenic functions. However, no significant differences were observed in the distribution of variants between groups of genes (chi square, p = 0,219) (Fig. 1). Details of the variants are available in **Supplementary Table 1**.

## Discussion

This research reports the results of post-mortem genetic testing performed through a comprehensive NGS analysis of genes associated with arrhythmogenic syndromes in a cohort of 76 SIDS cases.

As said, although the pathophysiological mechanism underlying SIDS is still unclear, it has been hypothesized that death is due to fatal arrhythmias dependent on the infant’s genetic background [22, 23]. Currently, a genetic cardiac predisposition that may have contributed to arrhythmogenic sudden infant death has been identified in several cohorts. This predisposition consists of rare/ultra-rare variants associated with inherited cardiac conditions (i.e., primary arrhythmia syndromes and structural cardiac diseases) [5, 24–28]. The main difficulties in identifying the actual burden of cardiac genetic predisposition in SIDS by comparing existing studies lie in the different types of post-mortem genetic testing performed, the panel of genes analyzed and the VAF cutoff considered.

In our study, NGS analysis was performed using a custom resequencing panel of selected genes related to SCD. Indeed, there is a well-established consensus that about 10–15% of SIDS are due to pathogenic mutations in cardiac channelopathy-associated genes [5, 9, 29, 30]. Moreover, we also considered genes associated with cardiomyopathies because alterations in structural proteins can be responsible for arrhythmic events even in absence of macro/microscopic changes. In our study, variants with a VAF < 0,1% were considered rare. This not too strict initial cutoff was set up to avoid excluding functional potentially important variants involved in the pathogenesis of SIDS. Although filtering from the beginning with a lower allele frequency (VAF < 0.005%) might be useful to rule out variants that are too frequent in the general population to cause rare diseases, it might miss potentially important variants related to SIDS but too common to be detected with the applied threshold [9, 31, 32]. With our threshold value set, post-mortem genetic analysis detected 50 carriers of at least one rare genetic variant associated with SCD. PM2_Supporting item in the ACGM classification was considered then fulfilled with VAF < 0.004% for cardiomyopathies and < 0.001% for channelopathies. Additionally, there is currently a lack of specific recommendations or guidelines focused on variant interpretation in forensic medicine, which further complicates accurate genetic assessment in forensic contexts [33].

Our post-mortem genetic testing revealed that in 4 out of 76 cases (5.3%) at least one pathogenic (P) or likely pathogenic (LP) variant could be considered as a monogenic cardiac cause responsible for the sudden death of the infant. Indeed, in all these cases, previous forensic examinations including full autopsy, histopathological, toxicological and microbiological analysis were negative/inconclusive.

The variants *TTN*_ p.(Leu22480*) and *TTN*_c.32705-1G > A (rs1285884266) were identified in an infant who died without a clear cause of death after full forensic examination. Both affect the *TTN* for a myofilament with a key role in the transmission of and maintenance of resting tension during cardiac contraction [34, 35]. Pathogenic variants in *TTN* are primarily linked to DCM. Although approximately 25% of DCM cases are attributed to truncations in the A-band region of *TTN* [36], mutations in other regions are also observed. For example, among *TTN* mutations associated with DCM, Chauveau et al. [37] reported 29 nonsense changes (including three in the I-band, and 26 in the A-band), along with 17 frameshift mutations (three in the I-band, 14 in the A-band). Additionally, 18 mutations affected *TTN* splicing, and seven were missense mutations (three in the Z-line, three in the I-band and one in the M-line). Hence, in the current study, despite the variant (*TTN*_c.32705-1G > A) not being situated within the A-band region, it’s noteworthy that other regions of the *TTN* gene are expressed in the cardiac isoform and potentially relevant to DCM, thus subject to evaluation in the variant analysis. Moreover, for variant classification, we consistently consider all exons of *TTN*. In our report, we specify when an exon exhibits a low Percentage Spliced In (PSI), referencing the information available at https://www.cardiodb.org/titin/titin_transcripts.php. Both these *TTN* variants found in our study were previously reported in ClinVar database and AutoPVS1 tool assign a PVS1_Very strong under a DCM phenotype. Following ACMG standards, these variants have been classified as LP because are absent or rare in the general population (PM2_Supporting) and undergo loss of function (LOF) through nonsense-mediated decay (NMD) of mRNA (PVS1_Very strong). The carrier also hosted some VUS variants. In the newborn with the two variants in *TTN* gene, a segregation analysis would be highly recommended to determine the allele distribution that could be the explanation for such a severe phenotype.

The variant in the *MYL3* gene, p.(Val156Leu)—rs199474707, CM1412322- was identified in a one-day-old newborn girl. *MYL3* encodes for the cardiac isoform of myosin essential light chain (ELC) modulating heart contraction [38]. This variant is observed in 22 out of 146,180 alleles in gnomAD, with a PopMax filtered frequency of 0.001213%. As previously stated, we consider that an extremely rare variant in cardiomyopathies is defined when the PopMax filtered is lower than 0.004%. Thus, PM2_Supporting item in the ACGM classification was considered fulfilled in the current case. Therefore, despite its infrequent occurrence in the general population, it has been detected in several HCM patients (PS4_Supporting). It is located in a protein domain critical for protein function (PM1_Strong) in a context of HCM and its key role is confirmed by in silico tools, predicting a deleterious effect (PP3). Considering all data, it was classified as LP. Several reports identified the same rare variant in patients diagnosed with HCM. A multivariate analysis performed by *Wang *et al*.* revealed that people with multiple rare variants in sarcomere genes have a higher risk of SCD [39]. Thus, the phenotype spectrum associated with the variant p.(Val156Leu) can range from an initially macro/microscopically undetectable condition to profibrotic changes that, at an advanced stage, result in an overt left ventricular hypertrophy (LVH) assessed by imaging techniques. Studies in animal models have shown that pathways involved in fibrosis and collagen deposition are activated early, before LVH is detectable at gross or histological examination [11, 40–44]. Thus, as hypothesized by *Brion *et al. [11], it is possible that the disruption of sarcomeric activity itself in the earliest stages of the disease, may alter intracellular calcium homeostasis exposing the patient to life-threating arrhythmias (LTAs). These mechanisms could be particularly relevant in SIDS, where there is no visible phenotype as in most incipient forms of the disease. However, further studies are needed to ascertain the role of certain cardiac structural proteins in modulating cardiac electrical conduction. On the other hand, this close interaction between ion channels and structural proteins, has already been established for genes coding for proteins with both structural and arrhythmogenic functions (e.g., *SCN5A, RYR2, HCN4, AKAP9*).

The variant p.(Trp1684Phe) in *SCN5A* gene was found in a 3-day-old infant who died while sleeping. Abnormalities in the cardiac sodium channel gene (*SCN5A*) are mainly associated with BrS and LQTS, which are characterized by an inherited susceptibility to ventricular arrhythmias. However, some variants in this gene cause enlargement of cardiac chambers, resulting in DCM. It has been hypothesized that alterations in this sodium channel may disrupt interactions with the cytoskeleton and intercalar disc or, alternatively, alter the amount of intracellular Ca^+2^ causing impaired contraction and structural deformation [45, 46]. No gross or histopathological changes were observed in our case. This variant does not result in a frameshift mutation. Its protein-level effect is expected to resemble that of a missense variant. As previously mentioned, although variants in *SCN5A* may be also reported especially in LQTS, in our case, the variant detected in the infant was classified in the context of BrS due to its location within a domain known to be crucial for the proper functioning of the protein and associated with BrS (PM1_Strong). Moreover, this variant was nearly absent in the general population (PM2_Supporting) and reported only once in ClinVar in a patient diagnosed with BrS. The LP significance of this variant was also supported by PP4 criteria, which a patient’s phenotype or family history is highly specific for a disease with a single genetic etiology. The new international guidelines published in 2022 still maintain *SCN5A* gene as the only gene with a definitive association with Brugada Syndrome. The risk of ventricular arrhythmias in children with BrS is generally low but fever (particularly frequent in early childhood) is the most important precipitating factor [47]. Moreover, the risk of SCD in pediatric BrS patients appears to be inversely related to age [48]. This evidence may support the role of channelopathies in predisposing infants to the fatal arrhythmias as in SIDS, especially when certain circumstances occur.

Our post-mortem genetic analysis identified a variant in the *SLC22A5* gene, p.(Arg282Gln)—rs386134210, CM061983- in a young male infant. The detected variant was the only one of our cohort that was possible to classify as pathogenic according to the ACMG guidelines. Although the inheritance pattern is autosomal recessive, this variant has been classified as pathogenic, despite not being found in the homozygous or compound heterozygous form. Indeed, the presence of an additional P or LP variant *in trans* in the unanalyzed regions of this gene not covered by the custom panel performed in the current study should be considered. Therefore, an additional rare variant in the same gene is necessary to be considered causative of systemic primary carnitine deficiency disease. This variant characterized by a low allele frequency is more common in patients with Systemic primary carnitine deficiency (CDSP) than in the general population (PM2_Supporting; PS4_Supporting). In ClinVar database, it is reported several times as pathogenic significance. Functional in vitro studies [49] revealed the damaging effect on chinese hamster ovary (CHO) cells in comparison to controls (PS3_Strong). *SLC22A5* gene encodes for the plasmalemmal carnitine transporter and the related disease encompasses a broad clinical spectrum including metabolic decompensation, cardiomyopathy, hypoglycemic hypoketotic encephalopathy or absence of symptoms [50, 51]. Progressive -both dilated and hypertrophic- cardiomyopathy are observed mainly in childhood, while arrhythmic events with shortened QT interval are described in adult people [52]. Roussel et al. [53] observed that the shortening of the QT interval at continuous electrocardiographic monitoring correlated negatively with plasma carnitine concentration causing ventricular fibrillation. These findings strongly suggest that long-chain fatty acid β-oxidation may influence the morphology and the electrical function of the heart leading to unexpected sudden death.

All above mentioned P/LP variants were found in structural or structural/arrhythmogenic genes. Despite the apparent greater involvement of structural genes compared to other gene groups, the difference in the distribution of variants was not statistically significant (chi square, p = 0,219) (Fig. 1).

The results of our analysis revealed that 5.3% of our cases exhibited at least one potentially causative variant, which could account for sudden and unexpected infant death. These findings are consistent with similar studies, such as those conducted by *Tester *et al*.* [9], who obtained comparable results by identifying pathogenic or likely pathogenic variants in 4.3% of 419 SIDS cases, even if with a minor allele frequency < 0.00005. However, in other studies, this percentage may reach up to 34% when filtered with a higher allele frequency, typically < 1% [54].

The evidence reported in our study support the utility of post-mortem genetic testing in cases of SIDS. However, the main challenge of the most advanced and comprehensive postmortem genetic analyses lies not in the technical identification of variants but in the interpretation of pathogenicity, assigning the variant a causal role in the infant's death. The identification of a variant as pathogenic has important implications for the relatives, so the clinical translation of laboratory results should be carried out carefully and a co-segregation analysis in the family members is highly recommended. The importance of segregation analysis among family members is well recognized in the scientific literature, as highlighted by European recommendations [55] for investigating SCD in adults and similarly emphasized by Kotta et al. [56] concerning sudden infant and early childhood death. In the current study, upon receiving the genetic study results from the Institute of Legal Medicine, family members were informed about the opportunity for clinical and genetic testing at a specialized unit for familial cardiopathies.

## Conclusions

Our NGS analysis revealed that 5.3% of the 76 SIDS cases had at least one potentially causative variant that could explain the sudden and unexpected death. The findings of our study are consistent with previous studies performed on cohorts characterized by a significant number of SIDS cases, low allele frequency and in accordance with the stringent recommendations provided by the ACMG. However, the lack of uniformity among the existing studies prevents understanding the real burden of cardiac genetic predisposition in SIDS. Moreover, to date several of the variants identified are of uncertain significance and there is currently a lack of specific recommendations or guidelines focused on variant interpretation in forensic medicine. Further studies are needed to unravel the actual pathogenicity of these variants and to clarify the mechanisms underlying the close interaction between cardiac structural proteins and ion channels, resulting in the sudden unexpected fatal arrhythmias in infants without a detectable phenotype.

### Study limitations

Limitations to the current study include mainly the restricted availability of post-mortem radiological information (since post-mortem radiological investigation was indicated only in selected cases) and the lack of genetic testing in the close relatives of all deceased infants to perform a comprehensive genotype- phenotype co-segregation analysis in all the families. Furthermore, despite a very comprehensive NGS panel, there may be some rare variants in other genes not included in our panel. A whole-exome sequencing (WES) analysis would allow a more extensive data analysis to investigate several potentially pathogenic mechanisms underlying SIDS. However, although WES has a higher diagnostic yield, the assessment of variants’ pathogenicity remains the main issue to be addressed, especially in a forensic field where specific guidelines for variant interpretation are currently lacking.

## Supplementary Information

Below is the link to the electronic supplementary material.

Supplementary file1 (XLSX 22 KB)

## Data Availability

Data are avaiable upon reasonable request to the corresponding author.

## References

[1] Moon RY, Darnall RA, Feldman-Winter L et al (2016) SIDS and other sleep-related infant deaths: Updated 2016 recommendations for a safe infant sleeping environment. Pediatrics 138. 10.1542/peds.2016-2938 10.1542/peds.2016-2938 27940804

[2] Carlin RF, Moon RY (2017) Risk Factors, Protective Factors, and Current Recommendations to Reduce Sudden Infant Death Syndrome. JAMA Pediatr 171:175. 10.1001/jamapediatrics.2016.3345 27918760 10.1001/jamapediatrics.2016.3345

[3] Krous HF, Beckwith JB, Byard RW et al (2004) Sudden Infant Death Syndrome and Unclassified Sudden Infant Deaths: A Definitional and Diagnostic Approach. Pediatrics 114:234–238. 10.1542/peds.114.1.234 15231934 10.1542/peds.114.1.234

[4] Byard RW, Ranson D, Krous HF (2005) National Australian Workshop Consensus on the Definition of <SMALL>SIDS</SMALL> and Initiation of a Uniform Autopsy Approach to Unexpected Infant and Early Childhood Death. Forensic Sci Med Pathol 1:289–292. 10.1385/FSMP:1:4:289 25868449 10.1385/FSMP:1:4:289

[5] Baruteau AE, Tester DJ, Kapplinger JD et al (2017) Sudden infant death syndrome and inherited cardiac conditions. Nat Rev Cardiol 14:715–726 28880023 10.1038/nrcardio.2017.129

[6] Campuzano O, Allegue C, Sarquella-Brugada G et al (2014) The role of clinical, genetic and segregation evaluation in sudden infant death. Forensic Sci Int 242:9–15. 10.1016/j.forsciint.2014.06.007 25016126 10.1016/j.forsciint.2014.06.007

[7] Harrington CT, al Hafid N, Waters KA (2022) Butyrylcholinesterase is a potential biomarker for Sudden Infant Death Syndrome. 10.1016/j 10.1016/j.ebiom.2022.104041 PMC9092508 35533499

[8] van Norstrand DW, Ackerman MJ (2010) Genomic risk factors in sudden infant death syndrome. Genome Med 2:86. 10.1186/gm207 21122164 10.1186/gm207 PMC3016628

[9] Tester DJ, Wong LCH, Chanana P et al (2018) Cardiac Genetic Predisposition in Sudden Infant Death Syndrome. J Am Coll Cardiol 71:1217–1227. 10.1016/j.jacc.2018.01.030 29544605 10.1016/j.jacc.2018.01.030

[10] Sarquella-Brugada G, Campuzano O, Cesar S et al (2016) Sudden infant death syndrome caused by cardiac arrhythmias: only a matter of genes encoding ion channels? Int J Legal Med 130:415–420 26872470 10.1007/s00414-016-1330-7

[11] Brion M, Allegue C, Santori M et al (2012) Sarcomeric gene mutations in sudden infant death syndrome (SIDS). Forensic Sci Int 219:278–281. 10.1016/j.forsciint.2012.01.018 22361390 10.1016/j.forsciint.2012.01.018

[12] Sarquella-Brugada G, Cesar S, Zambrano MD et al (2018) Electrocardiographic Assessment and Genetic Analysis in Neonates: a Current Topic of Discussion. Curr Cardiol Rev 15:30–37. 10.2174/1573403x14666180913114806 10.2174/1573403X14666180913114806 PMC6367699 30210005

[13] Semsarian C, Ingles J (2016) Molecular autopsy in victims of inherited arrhythmias. J Arrhythm 32:359–365. 10.1016/j.joa.2015.09.010 27761159 10.1016/j.joa.2015.09.010 PMC5063264

[14] Richards S, Aziz N, Bale S et al (2015) Standards and guidelines for the interpretation of sequence variants: a joint consensus recommendation of the American College of Medical Genetics and Genomics and the Association for Molecular Pathology. Genet Med 17:405–424. 10.1038/gim.2015.30 25741868 10.1038/gim.2015.30 PMC4544753

[15] Bajanowski T, Vege Å, Byard RW et al (2007) Sudden infant death syndrome (SIDS)—Standardised investigations and classification: Recommendations. Forensic Sci Int 165:129–143. 10.1016/j.forsciint.2006.05.028 16806765 10.1016/j.forsciint.2006.05.028

[16] (2013) Specific recommendations for the unification of judicial autopsies at the Institute of Legal Medicine of Catalonia. https://repositori.justicia.gencat.cat/bitstream/handle/20.500.14226/637/recommendations_judicial_autopsies.pdf?sequence=1&isAllowed=y

[17] Adzhubei IA, Schmidt S, Peshkin L et al (2010) A method and server for predicting damaging missense mutations. Nat Methods 7:248–249. 10.1038/nmeth0410-248 20354512 10.1038/nmeth0410-248 PMC2855889

[18] Choi Y, Sims GE, Murphy S et al (2012) Predicting the Functional Effect of Amino Acid Substitutions and Indels. PLoS ONE 7:e46688. 10.1371/journal.pone.0046688 23056405 10.1371/journal.pone.0046688 PMC3466303

[19] Schwarz JM, Cooper DN, Schuelke M, Seelow D (2014) MutationTaster2: mutation prediction for the deep-sequencing age. Nat Methods 11:361–362. 10.1038/nmeth.2890 24681721 10.1038/nmeth.2890

[20] Harris PA, Taylor R, Thielke R et al (2009) Research electronic data capture (REDCap)—A metadata-driven methodology and workflow process for providing translational research informatics support. J Biomed Inform 42:377–381. 10.1016/j.jbi.2008.08.010 18929686 10.1016/j.jbi.2008.08.010 PMC2700030

[21] Harris PA, Taylor R, Minor BL et al (2019) The REDCap consortium: Building an international community of software platform partners. J Biomed Inform 95:103208. 10.1016/j.jbi.2019.103208 31078660 10.1016/j.jbi.2019.103208 PMC7254481

[22] Evans A, Bagnall RD, Duflou J, Semsarian C (2013) Postmortem review and genetic analysis in sudden infant death syndrome: an 11-year review. Hum Pathol 44:1730–1736. 10.1016/j.humpath.2013.01.024 23623143 10.1016/j.humpath.2013.01.024

[23] Grassi S, Vidal MC, Campuzano O et al (2021) Sudden Death without a Clear Cause after Comprehensive Investigation: An Example of Forensic Approach to Atypical/Uncertain Findings. Diagnostics 11:886. 10.3390/diagnostics11050886 34067575 10.3390/diagnostics11050886 PMC8156818

[24] Campuzano O, Beltramo P, Fernandez A et al (2018) Molecular autopsy in a cohort of infants died suddenly at rest. Forensic Sci Int Genet 37:54–63. 10.1016/j.fsigen.2018.07.023 30086531 10.1016/j.fsigen.2018.07.023

[25] Neubauer J, Lecca MR, Russo G et al (2017) Post-mortem whole-exome analysis in a large sudden infant death syndrome cohort with a focus on cardiovascular and metabolic genetic diseases. Eur J Hum Genet 25:404–409. 10.1038/ejhg.2016.199 28074886 10.1038/ejhg.2016.199 PMC5386419

[26] Santori M, Blanco-Verea A, Gil R et al (2015) Broad-based molecular autopsy: a potential tool to investigate the involvement of subtle cardiac conditions in sudden unexpected death in infancy and early childhood. Arch Dis Child 100:952–956. 10.1136/archdischild-2015-308200 26272908 10.1136/archdischild-2015-308200

[27] Mates J, Mademont-Soler I, Fernandez-Falgueras A et al (2020) Sudden Cardiac Death and Copy Number Variants: What Do We Know after 10 Years of Genetic Analysis? Forensic Sci Int Genet 47:102281. 10.1016/j.fsigen.2020.102281 32248082 10.1016/j.fsigen.2020.102281

[28] Vallverdú-Prats M, Alcalde M, Sarquella-Brugada G et al (2021) Rare Variants Associated with Arrhythmogenic Cardiomyopathy: Reclassification Five Years Later. J Pers Med 11:162. 10.3390/jpm11030162 33652588 10.3390/jpm11030162 PMC7996798

[29] Tester DJ, Ackerman MJ (2014) Cardiac Channelopathies and the Molecular Autopsy. Forensic Pathology of Infancy and Childhood. Springer, New York, New York, NY, pp 899–942

[30] Cannon SC (2018) Skeletal muscle channelopathy: a new risk for sudden infant death syndrome. Lancet 391:1457–1458. 10.1016/S0140-6736(18)30477-X 29605428 10.1016/S0140-6736(18)30477-X

[31] Köffer J, Scheiper-Welling S, Verhoff MA et al (2021) Post-mortem genetic investigation of cardiac disease–associated genes in sudden infant death syndrome (SIDS) cases. Int J Legal Med 135:207–212. 10.1007/s00414-020-02394-x 32789579 10.1007/s00414-020-02394-x PMC7782403

[32] Grassi S, Campuzano O, Coll M et al (2020) Genetic variants of uncertain significance: How to match scientific rigour and standard of proof in sudden cardiac death? Leg Med 45:101712. 10.1016/j.legalmed.2020.101712 10.1016/j.legalmed.2020.101712 32361481

[33] Martínez-Barrios E, Grassi S, Brión M et al (2023) Molecular autopsy: Twenty years of post-mortem diagnosis in sudden cardiac death. Front Med (Lausanne) 10. 10.3389/fmed.2023.1118585 10.3389/fmed.2023.1118585 PMC9950119 36844202

[34] LeWinter MM, Granzier H (2010) Cardiac Titin. Circulation 121:2137–2145. 10.1161/CIRCULATIONAHA.109.860171 20479164 10.1161/CIRCULATIONAHA.109.860171 PMC2905226

[35] Itoh-Satoh M, Hayashi T, Nishi H et al (2002) Titin Mutations as the Molecular Basis for Dilated Cardiomyopathy. Biochem Biophys Res Commun 291:385–393. 10.1006/bbrc.2002.6448 11846417 10.1006/bbrc.2002.6448

[36] Gigli M, Begay RL, Morea G et al (2016) A Review of the Giant Protein Titin in Clinical Molecular Diagnostics of Cardiomyopathies. Front Cardiovasc Med 3. 10.3389/fcvm.2016.00021 10.3389/fcvm.2016.00021 PMC4954824 27493940

[37] Chauveau C, Rowell J, Ferreiro A (2014) A Rising Titan: TTN Review and Mutation Update. Hum Mutat 35:1046–1059. 10.1002/humu.22611 24980681 10.1002/humu.22611

[38] Osborn DPS, Emrahi L, Clayton J et al (2021) Autosomal recessive cardiomyopathy and sudden cardiac death associated with variants in MYL3. Genet Med 23:787–792. 10.1038/s41436-020-01028-2 33288880 10.1038/s41436-020-01028-2 PMC8026398

[39] Wang J, Wang Y, Zou Y et al (2014) Malignant effects of multiple rare variants in sarcomere genes on the prognosis of patients with hypertrophic cardiomyopathy. Eur J Heart Fail 16:950–957. 10.1002/ejhf.144 25132132 10.1002/ejhf.144

[40] Morita H, Larson MG, Barr SC et al (2006) Single-Gene Mutations and Increased Left Ventricular Wall Thickness in the Community. Circulation 113:2697–2705. 10.1161/CIRCULATIONAHA.105.593558 16754800 10.1161/CIRCULATIONAHA.105.593558

[41] Ho CY, Abbasi SA, Neilan TG et al (2013) T1 Measurements Identify Extracellular Volume Expansion in Hypertrophic Cardiomyopathy Sarcomere Mutation Carriers With and Without Left Ventricular Hypertrophy. Circ Cardiovasc Imaging 6:415–422. 10.1161/CIRCIMAGING.112.000333 23549607 10.1161/CIRCIMAGING.112.000333 PMC3769196

[42] de Marvao A, McGurk KA, Zheng SL et al (2021) Phenotypic Expression and Outcomes in Individuals With Rare Genetic Variants of Hypertrophic Cardiomyopathy. J Am Coll Cardiol 78:1097–1110. 10.1016/j.jacc.2021.07.017 34503678 10.1016/j.jacc.2021.07.017 PMC8434420

[43] Grassi S, Campuzano O, Coll M et al (2021) Update on the Diagnostic Pitfalls of Autopsy and Post-Mortem Genetic Testing in Cardiomyopathies. Int J Mol Sci 22:4124. 10.3390/ijms22084124 33923560 10.3390/ijms22084124 PMC8074148

[44] Berge KE, Leren TP (2014) Genetics of hypertrophic cardiomyopathy in Norway. Clin Genet 86:355–360. 10.1111/cge.12286 24111713 10.1111/cge.12286

[45] Wilde AAM, Amin AS (2018) Clinical Spectrum of SCN5A Mutations. JACC Clin Electrophysiol 4:569–579. 10.1016/j.jacep.2018.03.006 29798782 10.1016/j.jacep.2018.03.006

[46] Zaklyazminskaya E, Dzemeshkevich S (2016) The role of mutations in the SCN5A gene in cardiomyopathies. Biochim Biophys Acta (BBA) - Mol Cell Res 1863:1799–1805. 10.1016/j.bbamcr.2016.02.014 10.1016/j.bbamcr.2016.02.014 26916278

[47] Probst V, Denjoy I, Meregalli PG et al (2007) Clinical Aspects and Prognosis of Brugada Syndrome in Children. Circulation 115:2042–2048. 10.1161/CIRCULATIONAHA.106.664219 17404158 10.1161/CIRCULATIONAHA.106.664219

[48] Andorin A, Behr ER, Denjoy I et al (2016) Impact of clinical and genetic findings on the management of young patients with Brugada syndrome. Heart Rhythm 13:1274–1282. 10.1016/j.hrthm.2016.02.013 26921764 10.1016/j.hrthm.2016.02.013

[49] Filippo CA di S, Pasquali M, Longo N (2006) Pharmacological rescue of carnitine transport in primary carnitine deficiency. Hum Mutat 27:513–523. 10.1002/humu.20314 10.1002/humu.20314 16652335

[50] Li F-Y, El-Hattab AW, Bawle EV et al (2010) Molecular spectrum of SLC22A5 (OCTN2) gene mutations detected in 143 subjects evaluated for systemic carnitine deficiency. Hum Mutat 31:E1632–E1651. 10.1002/humu.21311 20574985 10.1002/humu.21311

[51] El-Hattab AW, Scaglia F (2015) Disorders of carnitine biosynthesis and transport. Mol Genet Metab 116:107–112. 10.1016/j.ymgme.2015.09.004 26385306 10.1016/j.ymgme.2015.09.004

[52] Fu L, Huang M, Chen S (2013) Primary Carnitine Deficiency and Cardiomyopathy. Korean Circ J 43:785. 10.4070/kcj.2013.43.12.785 24385988 10.4070/kcj.2013.43.12.785 PMC3875693

[53] Roussel J, Labarthe F, Thireau J et al (2016) Carnitine deficiency induces a short QT syndrome. Heart Rhythm 13:165–174. 10.1016/j.hrthm.2015.07.027 26190315 10.1016/j.hrthm.2015.07.027

[54] Hertz CL, Christiansen SL, Larsen MK et al (2016) Genetic investigations of sudden unexpected deaths in infancy using next-generation sequencing of 100 genes associated with cardiac diseases. Eur J Hum Genet 24:817–822. 10.1038/ejhg.2015.198 26350513 10.1038/ejhg.2015.198 PMC4867441

[55] Fellmann F, van El CG, Charron P et al (2019) European recommendations integrating genetic testing into multidisciplinary management of sudden cardiac death. Eur J Hum Genet 27:1763–1773. 10.1038/s41431-019-0445-y 31235869 10.1038/s41431-019-0445-y PMC6870982

[56] Kotta M, Torchio M, Bayliss P et al (2023) Cardiac Genetic Investigation of Sudden Infant and Early Childhood Death: A Study From Victims to Families. J Am Heart Assoc 12. 10.1161/JAHA.122.029100 10.1161/JAHA.122.029100 PMC10547337 37589201

